# Framing Marketing Responses to National Regulation: The Four Ps in Transnational Corporate Political Discourse: Comment on "Understanding Marketing Responses to a Tax on Sugary Drinks: A Qualitative Interview Study in the United Kingdom, 2019"

**DOI:** 10.34172/ijhpm.2022.7618

**Published:** 2022-11-20

**Authors:** Kathrin Lauber

**Affiliations:** Global Health Policy Unit, School of Social and Political Science, University of Edinburgh, Edinburgh, UK.

**Keywords:** Obesity, Tax, Sugar-Sweetened Beverages, Lobbying

## Abstract

A growing evidence base indicates that sugar-sweetened beverage (SSB) taxes are an effective tool to help reduce excess sugar intake. The effects of SSB taxes and the mechanisms which underlie them, however, are dependent on a number of interrelated factors such as policy design and responses of industry and consumers. Forde and colleagues contribute to unpacking these mechanisms by exploring the way in which the UK’s Soft Drinks Industry Levy (SDIL) shaped the four Ps of soft drinks marketing: product, price, placement, and promotion. This commentary builds on the authors’ insights by connecting them to existing knowledge on corporate political activity and the commercial determinants of health (CDOH) more broadly. Specifically, I discuss the risk that an industry framing of regulation-induced marketing changes as a voluntary step towards corporate responsibility undermines the need for government intervention to address obesity in other contexts and countries. I conclude by arguing that the public health community would benefit from considering marketing responses to regulation alongside industry narratives about these changes.

## Introduction

 Sugar-sweetened beverage (SSB) taxes have emerged as effective and increasingly popular instruments to increase prices and lower sales of SSBs. Although not enough time has passed to clearly demonstrate long-term health impacts, SSB taxes, like all population-level interventions, are not intended as a ‘one-policy fix’ but rather part of wider efforts to address a complex problem. SSB taxation can impact health through multiple pathways such as price changes, public awareness about health effects of sugar, and product reformulation [sugar reduction that often involves replacing some or all sugar with non-nutritive sweeteners]. These impacts vary by tax design; tiered designs, for instance, have consistently led to product reformulation whilst public perception of SSB is likely to be more desirably affected where a tax is introduced with an explicit health objective and clear definition of the tax scope.^1,2^

 The two-tiered Soft Drinks Industry Levy (SDIL) was introduced in 2016 as a measure to address the UK’s high obesity rates and implemented in 2018 after a two-year transition period. Evaluation results showed that many manufacturers reduced the sugar content of their SSBs and some of the levy’s cost was passed on to consumer in the form of higher prices.^3^ Compared to pre-levy trends, sugar in purchased soft drinks fell by 10% per household while the overall volume of soft drinks consumed did not change.^4^

 Forde and colleagues^5^ make an important contribution to our understanding of potential pathways for policy impact with a nuanced analysis of the decision-making underlying marketing responses to the SDIL. Specifically, the authors explore mechanisms behind marketing responses across the ‘four Ps’ to encompass impacts on *product* (reformulation) and *price* as well as *placement* and *promotion* which are less commonly discussed in the context of fiscal policy. Drawing on interviews with experts from industry, civil society, and academia, the authors present a theoretical framework of marketing decision-making which accounts for the role of internal and external context in interaction with purchasing responses, suggesting that a better understanding of such processes can help predict company-level marketing responses and support policy-makers in designing more effective policies.

 In this commentary, I seek to expand on the authors’ findings by discussing their contribution on marketing decision-making in light of what we know about industry political practices. For this purpose, I adopt a commercial determinants of health (CDOH) lens that views corporate practices as deeply interconnected. The CDOH are defined as “the social, political, and economic structures, norms, rules, and practices by which business activities designed to generate profits and increase market share influence patterns of health, disease, injury, disability, and death within and across populations.”^6^ Within this wider picture, political practices – ranging from direct lobbying to more indirect activities such as creating a sense of broad support for the industry position through seemingly independent front groups – are crucial to disseminating narratives that position effective regulation of unhealthy commodities as an unnecessary or inappropriate solution to public health issues like obesity. A CDOH approach draws attention to the reality that marketing and political practices fundamentally serve the same goal. Further, marketing is used to change and maintain not only the social norms and desirability of products, but also of firms and brands. It is a pathway to shape the public (and by extension, political) discourse surrounding those products, as well as the reputation and legitimacy of brands and companies.^7^ This renders marketing an important tool for the (de-)problematisation of products or practices which has implications for whether and how governments should intervene.

 Although Forde and colleagues briefly mention that non-market activities such as lobbying can shape the position of a product alongside marketing, this largely lies outside the scope of their analysis. Fiscal interventions, however, have been the subject of much political attention within the soft drinks industry and sustained pushback a strikingly common response to SSB tax proposals or the threat thereof.^8^ A leaked 2016 Coca-Cola Company “public policy risk matrix,” for instance, maps policies across their expected business impact and likelihood to materialise, highlighting SSB taxes as particularly threatening and thus a major lobbying target.^9^

 Below I seek to link what we know about marketing and political practices in the SDIL context by unpacking the way in which marketing changes – real or rhetorical – have featured in corporate political discourse. For this purpose, I do not consider it helpful to focus on the soft drinks industry as a whole; instead, I concentrate on transnational producers and major business associations. There are two reasons for this: *firstly*, existing evidence suggests that this particular set of industry actors is most commonly implicated in the pushback governments encounter when considering SSB taxes; *secondly*, the transnational nature of the companies that dominate the global soft drinks market brings with it a particular set of implications closer to that of market leaders in other sectors than that of small beverage manufacturers. The latter point is rooted in the idea that market power readily translates into political power, in that it comes with resources and structural advantages which enable marketing and political efforts that lie beyond the reach of most businesses, particularly in highly concentrated industries like soft drinks.

## Marketing Changes Within Corporate Political Discourse – Shaping Perceptions Around Who Changed What and Why

 The framing of industry actions and their impacts, is a powerful tool that shapes how they are interpreted. The intersection between marketing and political discourse reveals valuable insights into the ways in which changes to the four Ps may be used in efforts shape policy in line with industry preferences. Specifically, I argue that reframing product reformulation as industry-led rather than a response to government intervention may contribute to misperceptions that voluntary reformulation initiatives are a desirable alternative to regulation.

 In addition to forming an important part of the industry response to SSB taxation, marketing changes are introduced in anticipation of policy change. While we can assume that this reflects, in part, a legitimate desire to adapt early to upcoming policy change, it is probable that a further motivation is the prevention of regulation. Among common industry arguments against the introduction of SSB taxes and other dietary public health regulation, one core narrative of *policy redundancy* leans strongly on existing actions or commitments to position government intervention as unnecessary. In the lead-up to the SDIL adoption, this included framing the levy and industry-driven voluntary measures such as reformulation and advertising codes as an ‘either or’ scenario by arguing that the regulatory burdens imposed by the levy will “make it more difficult to continue investing in the changes” underway.^10^

 Once a policy has been implemented, whether and how it has worked in one country is likely to shape actions by policy-makers seeking to address a similar issue elsewhere. Such* policy learning *from national and international experience is an important factor in the diffusion of SSB taxes.^11^ The United Kingdom represents an important reference country in this context, as one of the earliest to adopt an SSB tax with an explicit health objective. The role of corporate marketing responses within processes of policy learning is twofold:* firstly*, as Forde and colleagues highlight, marketing responses can influence a policy’s effectiveness; *secondly*, they can be presented or perceived as voluntary actions, particularly where the link to SSB taxation is less evident.

 Following evaluation results which indicate that the announcement of the levy sparked significant reformulation and a major reduction in high-sugar drinks,^3^ the British Soft Drinks Association and transnational producers remain keen to position this development as a continuation of voluntary sugar reduction efforts^10,12^ and an indicator that industry is stepping up to its role in helping tackle obesity, rather than as an intended response to the SDIL.^12^ While many product changes were indeed accelerated rather than prompted by the introduction of the levy, it is notable that the sugar reduction efforts of branded SSBs were lagging far behind retailers’ own-brand products prior to the SDIL announcement.^3^ Thus, and further to Forde and colleagues’ findings, I argue that the *perception* as to why marketing changes are happening is relevant because it has the potential to feed into a common framing of the companies which manufacture and sell unhealthy products as a part of the solution to the public health issues that they help create. Undoubtedly, the notion that companies can contribute to solving these issues appeals in theory, yet in practice, it can delay much needed public health action where voluntary measures are adopted instead of – not in addition to – regulation.^8,13^ In addition to questions about their effectiveness, partnership approaches seeking to encourage change within the industry by pursuing a ‘common ground’ between profit and health raise major governance issues where interests directly conflict.^14^ The public health community should be vigilant that marketing changes, particularly those that are less clearly connected to SSB taxation, may be framed as an indicator (and promise) of corporate responsibility rather than a policy response, potentially leading to an expectation that corporations will behave similarly elsewhere even in the absence of regulation.

 Lastly, compliance with and outward endorsement of regulatory measures should not be taken to equal actual support. Although the point has been made that companies favour interventions such as taxation because they level the playing field^15^ and this may well apply to some actors within the industry, a public display of policy acceptance should not necessarily be interpreted as actual acceptance as long as the same companies continue to oppose such measures internationally. As Forde and colleagues note, companies desire to be seen to “do the right thing”; this offers an alternative interpretation of a strategic shift in public communication following policy adoption, aligned with changing political opportunities rather than an internal positions. The overturning of Norway’s long-standing SSB tax in 2021, following pressure from business groups representing many of the companies also active in the United Kingdom, shows that retrenchment is a risk.^8^ On the other hand, identifying parts of the industry that are (or could be) genuinely supportive of policy change could be important for coalitions seeking to support the introduction of SSB taxes and other regulatory measures aimed at improving our food environment.

## Conclusion

 For those of us working primarily on one strand of the CDOH, it is important to consider how the four Ps of marketing interact with other mechanisms and practices. How companies draw on their marketing activities in international political spaces matters, particularly in light of a continuing lack of adequate action on healthier food systems globally. Added sugar intake is decreasing in many high-income countries but continues to rise in many low- and middle-income countries.^16^ Framing actions taken under threat of regulation as responsible corporate behaviour in some countries while simultaneously continuing to drive sales of unhealthy products in in others, where this threat is less acute, amounts to a double standard.

 Further, the scope for marketing responses is shifting constantly and in more concrete terms than the scope for political activity. A growing push within the dietary public health community to move beyond individual nutrients or ingredients and focus on health harms associated with ultra-processed products, for instance, challenges the idea of reformulation with non-nutritive sweeteners as a desirable policy outcome. Beverage reformulation is, moreover, significantly more feasible than food reformulation, which means that this particular marketing response would also be limited were fiscal measures extended to food as proposed, for example, in the 2021 National Food Strategy independent review. While a food tax is not currently being considered and the future of planned restrictions on advertising of products high in far, sugar, and salt remains uncertain, new restrictions on these products came into force in October 2022. Among these fast-shifting opportunity structures, it will be important for the public health community to consider and critically examine both industry marketing changes and accompanying narratives within their wider political and market context.

## Acknowledgements

 I would like to thank Eleanor Brooks and Dan Hunt for their feedback on earlier versions of this article, and the three anonymous reviewers for their thoughtful comments.

## Ethical issues

 Not applicable.

## Competing interests

 Author declares that she has no competing interests.

## Author’s contribution

 KL is the single author of the paper.
